# Urban Environments Promote Adaptation to Multiple Stressors

**DOI:** 10.1111/ele.70074

**Published:** 2025-02-19

**Authors:** Elizabeta Briski, Louisa Langrehr, Syrmalenia G. Kotronaki, Alena Sidow, Cindy Giselle Martinez Reyes, Antonios Geropoulos, Gregor Steffen, Nora Theurich, James W. E. Dickey, Jasmin C. Hütt, Phillip J. Haubrock, Ismael Soto, Antonín Kouba, Ross N. Cuthbert

**Affiliations:** ^1^ GEOMAR Helmholtz‐Zentrum für Ozeanforschung Kiel Kiel Germany; ^2^ Carl von Ossietzky Universität Oldenburg Oldenburg Germany; ^3^ Department of Biological Sciences Lehigh University Bethlehem Pennsylvania USA; ^4^ Faculty of Science and Technology Biology Department University of Crete, Marine Ecology Lab Crete Greece; ^5^ Christian‐Albrechts‐Universität Zu Kiel Christian‐Albrechts‐Platz 4 Kiel Germany; ^6^ Berlin‐Brandenburg Institute of Advanced Biodiversity Research Berlin Germany; ^7^ Institute of Biology Freie Universität Berlin Berlin Germany; ^8^ Leibniz Institute of Freshwater Ecology and Inland Fisheries (IGB) Berlin Germany; ^9^ Faculty of Fisheries and Protection of Waters, South Bohemian Research Center of Aquaculture and Biodiversity of Hydrocenoses University of South Bohemia in České Budějovice České Budějovice Czech Republic; ^10^ Center for Applied Mathematics and Bioinformatics, Department of Mathematics and Natural Sciences Gulf University for Science and Technology Hawally Kuwait; ^11^ Institute for Global Food Security, School of Biological Sciences Queen's University Belfast Belfast UK

**Keywords:** aquatic ecosystem, environmental change, land use, pCO_2_, salinity, temperature

## Abstract

Anthropogenic activities have drastically changed environmental conditions worldwide, negatively impacting biodiversity and ecosystem services. At the same time, the majority of the human population lives in urban areas that are greatly altered from natural habitats. Nevertheless, many species thrive in these urban environments. To improve our knowledge of evolution and adaptation in these anthropogenically impacted habitats, we conducted the widest series of stress experiments to date with three marine taxa: one mussel and two gammarid species. We compared intraspecific populations from protected and human‐altered habitats to determine their tolerance to salinity, temperature and partial pressure of CO_2_ in water (pCO_2_) regimes. Populations from impacted habitats typically outperformed protected habitat populations, with individuals from the most impacted habitat being the most robust. We propose that urban populations are adapting to life in disturbed environments—this adaptation concurrently promotes more resilient rescue populations but potentially confers increased invasion risk from non‐native species.

## Graphical abstract

To improve our knowledge of evolution and adaptation in urban and anthropogenically impacted habitats, we conducted the widest series of stress experiments to date with three marine taxa: one mussel *Mytilus* sp. and two gammarid species *Gammarus locusta* and *G. salinus* . Populations from impacted habitats typically outperformed protected habitat populations, with individuals from the most impacted habitat being the most robust. We propose that urban populations are adapting to life in disturbed environments—this adaptation concurrently promotes more resilient rescue populations but potentially confers increased invasion risk from non‐native species.

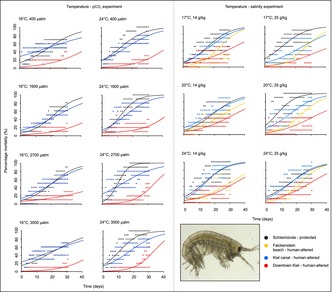

## 1 Introduction

The Earth and its environments are being rapidly changed by increasing human populations and economic development (Hawkins 2012). Anthropogenic activities, including burning of fossil fuels and deforestation, among others, have triggered climate change, pollution, soil erosion and habitat destruction (Hawkins 2012; IPCC 2014). The introduction of non‐native species (i.e. species moved by human activity outside their native region) is also prominent due to the increasing global trade and transport of materials (Hulme 2009; Sardain, Sardain, and Leung 2019). Subsequently, these changes have dramatically affected numerous ecosystems worldwide, leading to biotic homogenization, decreases in biodiversity, and occasionally, to losses of ecosystem functioning and services (Olden et al. 2004; Hawkins 2012; IPCC 2014; Linders et al. 2019; Pyšek et al. 2020).

More than half of the global human population presently lives in urban areas, with these numbers expected to double by 2050 (Johnson and Munshi‐South 2017; United Nations Statistics Division 2017). This urbanised world consists of dense settlements made of buildings, roads, and other supporting infrastructures, with urban habitats significantly altered from natural ones (Johnson and Munshi‐South 2017). As a consequence of rising urbanisation, environments in cities exhibit altered microclimates, which trend towards higher temperatures, alongside elevated air, water, noise, and light pollution (LLUR 2001; Grimm et al. 2008; Nikulina, Polovodova, and Schönfeld 2008). They also experience increased habitat fragmentation (Dobbs, Nitschke, and Kendal 2017), lower diversity and abundance of native species, high numbers of non‐native species (Aronson et al. 2014; McKinney 2008), and lower phylogenetic diversity within communities (Knapp et al. 2012; Sol et al. 2017). Still, urban environments are successfully exploited by many non‐human species (Johnson and Munshi‐South 2017; Perry and Gottert 2024). Pigeons, peregrine falcons, ducks, carps and goldfish are just some of the examples of those that thrive in urban habitats.

While rates of evolution had been deemed to be too slow to have relevance to urbanisation, recent research across a wide variety of taxa has demonstrated that organisms can indeed evolve rapidly in response to urban environments (Alberti et al. 2017; Johnson and Munshi‐South 2017; Caizergues, Grégoire, and Charmantier 2018; Santangelo, Rivkin, and Johnson 2018; Thompson, Rieseberg, and Schluter 2018; Borden and Flory 2021; Perry and Gottert 2024). Evolutionary changes have been observed in as little as two generations (Kinnison and Hendry 2001), and can include behavioural, morphological and physiological adaptations, or even speciation, in response to altered environmental conditions, novel resources and different physical habitats, among others (Giraudeau et al. 2014; Winchell et al. 2016; Martin et al. 2019; Borden and Flory 2021; Ålund et al. 2024). These changes may be particularly rapid in urban environments, where significant alterations to numerous abiotic and biotic factors are simultaneous and often predictable. Therefore, studies of adaptation in urban environments may be ideally placed for testing wider evolutionary theory across taxa (McKinney 2002; Grimm et al. 2008), particularly in the context of multiple stressors (Todgham, Schulte, and Iwama 2005). In turn, understanding the adaptation potential of diverse taxa, as well as ecosystem resilience to anthropogenic stressors, urbanisation, and global change, are of paramount importance to inform conservation actions, even in natural habitats.

In the last two decades, whereas research into the effects of global change on ecosystems has grown substantially, eco‐evolutionary dynamics in urban environments have been neglected outside of the terrestrial realm (Johnson and Munshi‐South 2017; Santangelo, Rivkin, and Johnson 2018; Thompson, Rieseberg, and Schluter 2018; Alter et al. 2020; Borden and Flory 2021; Perry and Gottert 2024). Marine ecosystems have been particularly overlooked in terms of urban evolution, but provide exceptional model systems considering the presence of multiple stressors and clear disturbance gradients (e.g. harbours versus protected areas). To improve our knowledge on evolution and adaptation potential of taxa in urban and anthropogenically impacted habitats, we conducted a series of laboratory experiments among populations of one mussel and two gammarid species collected at protected and human‐altered habitats in the Baltic Sea, to determine their tolerance (i.e. plasticity) to diverse levels of salinity, temperature and pCO_2_ (i.e. partial pressure of CO_2_ in water). Taking into account the concurrent evolution of adaptation to heterologous stressors (Todgham, Schulte, and Iwama 2005), the overall aim of this study was to test whether populations inhabiting human‐altered habitats are better adapted to anthropogenic stressors, including climate change, than populations from protected ones. We tested the null hypothesis that there is no difference in stress resistance between populations inhabiting human‐altered and protected habitats.

## 2 Materials and Methods

### 2.1 Experimental Organisms and Collection Sites

**FIGURE 1:**
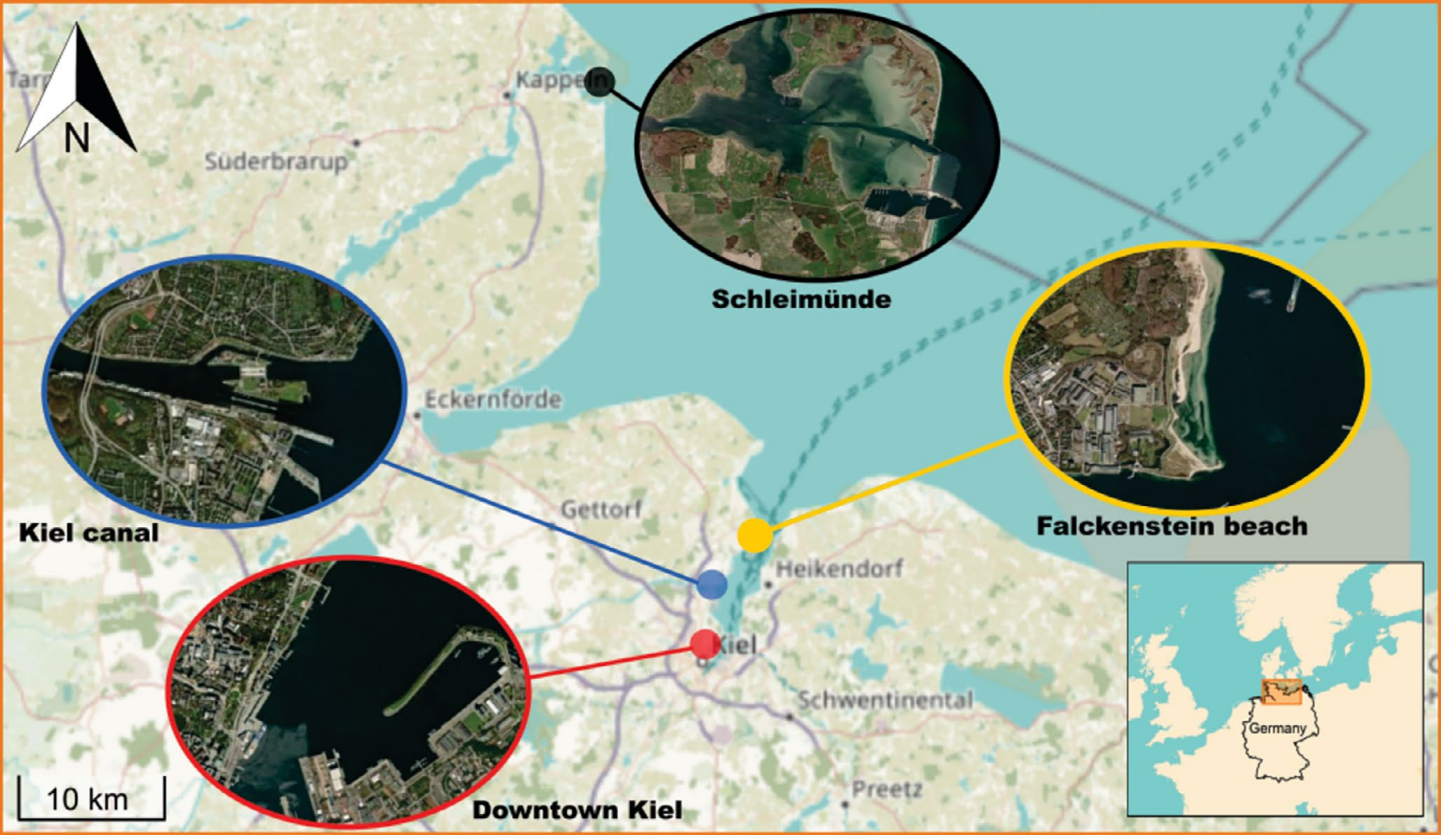
Sampling locations used in the study, where individuals of species populations were collected. Schleimünde served as the protected site, whereas Falckenstein beach, Kiel canal and downtown Kiel served as human‐altered sites.

Individuals of populations of one mussel (i.e. *Mytilus* sp.) and two gammarid species (i.e. *Gammarus locusta* and *G. salinus* ) were collected in protected and human‐altered (i.e. impacted) habitats in the Baltic Sea in Germany (Figure 1). Only hybrids of *M. edulis* and *Mytilus trossulus* are present at our sampling locations (Knöbel et al. 2021); therefore, we refer to the mussel species used as *Mytilus* sp. In the case of gammarids, each individual was morphologically identified according to Köhn and Gosselck (1989) and Zettler and Zettler (2017). All three taxa used in the experiments are native to the Baltic Sea (Springer and Crespi 2007; Gaitán‐Espitia et al. 2016; Cuthbert et al. 2020; Briski et al. 2024). While both gammarid species (alongside ~99% of global biodiversity) do not yet have an invasion record (Cuthbert et al. 2020; Briski et al. 2024), the mussel *M. edulis* is non‐native in Southern Europe, Asia and North America (Springer and Crespi 2007; Gaitán‐Espitia et al. 2016; Briski et al. 2024); *M. trossulus* is non‐native in the Black Sea (Briski et al. 2024). Though, we emphasise that *Mytilus* spp. taxonomy and distribution are complex due to their cryptic morphology and the frequent occurrence of hybridization between species (Springer and Crespi 2007; Gaitán‐Espitia et al. 2016). The protected habitat, Schleimünde (Maasholm), has been a nature reserve since 1972 (Verein Jordsand zum Schutze der Seevögel und der Natur e.V., 2019). According to its ‘EU Water Framework Directive’ status, the Schlei system is in a good chemical condition (LLUR 2001; Alpert et al. 2015). Though, we emphasise that there were no chemical concentrations reported. The human‐altered habitat was the Kiel fjord, with three locations sampled, depending on the species: (i) downtown Kiel; (ii) Kiel canal; and (iii) Falckenstein beach (Figure 1). Downtown Kiel is a completely artificial habitat with concrete structures, while the entire Kiel fjord, including downtown Kiel, is heavily impacted by the shipping industry and tourism, and exposed to extensive pollution including heavy metals such as copper, zinc, tin, lead, as well as tributyltin (LLUR 2001; Nikulina, Polovodova, and Schönfeld 2008). The levels of copper, zinc, tin and lead were higher deeper in the Fjord, with values increasing from 40, 80, 4 and 40 mg/kg at Falckenstein beach to 100, 240, 16 and 100 mg/kg at downtown Kiel, respectively (Nikulina, Polovodova, and Schönfeld 2008).

Individuals of at least two populations of each species were collected: one population in the protected habitat and one or more in a human‐altered habitat. In the case of *G. salinus* , three populations were sampled in human‐altered habitats alongside a protected one. In this case, we aimed to determine if there was a difference in performance among populations adapted to various human‐altered habitats along a perceived gradient of anthropogenic influence. Species were collected from May 2016 until January 2021, depending on when a particular species/population experiment was conducted as well as our sampling logistics. In the majority of cases, experimentation was in the same year and season for population comparisons from an individual species (Table S1). After collection, the animals were transported to the laboratories at GEOMAR Helmholtz‐Zentrum für Ozeanforschung Kiel in their ambient water and acclimatised for at least 2 weeks before the experiments commenced. During the acclimatisation period, animals were kept in their ambient water in 56 L glass aquaria in the laboratory environment (separately per species/population; Table S1). Each aquarium was aerated through a sponge filtration system to maintain water quality. The light regime was 12:12 h light and dark. Mussels were fed every 3 days with commercial live marine phytoplankton (Premium Reef Blend, Sustainable Aquatics), while gammarids were fed weekly with a mixture of commercial crustacean food (Tetra Mix, Tetra Crusta, and Dr. Shrimp Healthy).

### 2.2 Experimental Design

Two different types of experiments were conducted for each population of each species, with two crossed stressors: (i) temperature—pCO_2_ (i.e. partial pressure of CO_2_ in water); and (ii) temperature—salinity (Table 1). In the case of the temperature—pCO_2_ experiments, a fully crossed factorial design consisting of two temperature levels and four pCO_2_ levels was performed (Table 1). The temperature—salinity experiments consisted of three temperature and two salinity levels (Table 1). The temperature and pCO_2_ values were based on current and future predicted values for the Baltic Sea, as well as occasional measurements in Kiel fjord due to heat waves and seasonal upwelling events (Thomsen et al. 2012; Pansch et al. 2018; Meier et al. 2022). The lower salinity value was based on the regularly measured salinity of the sampled locations, with the higher salinity constituting a rare but ecologically relevant level measured during winter months at the impacted habitats (Kazanavičiūtė et al. 2024; Briski, personal observations).

**TABLE 1:** Outline of experimental parameters used for (a) temperature—pCO_2_ *Mytilus* sp., *G. locusta* , and *G. salinus* , (b) temperature‐salinity—*Mytilus* sp. and (c) temperature‐salinity— *G. locusta* and *G. salinus* experiments.

(a) Temperature—pCO_2_ experiments—*Mytilus* sp., *G. locusta*, and *G. salinus*								
Treatment	Ambient	T1	T2	T3	T4	T5	T6	T7
Temperature (°C)	16	16	16	16	24	24	24	24
pCO_2_ (μatm)	400	1 600	2 700	3 500	400	1 600	2 700	3 500
(b) Temperature—salinity experiments—*Mytilus* sp.								
Treatment	Ambient	T1	T2	T3	T4	T5		
Temperature (°C)	16	20	25	16	20	25		
Salinity (g/kg)	16	16	16	27	27	27		
(c) Temperature—salinity experiments—*G. locusta* and *G. salinus*								
Treatment	Ambient	T1	T2	T3	T4	T5		
Temperature (°C)	17	20	24	17	20	24		
Salinity (g/kg)	14	14	14	25	25	25		

*Note:* The strength of the treatments are presented by the darkness of the shades.

In both types of these multiple‐stressor experiments with factorial stressor arrangements, following the acclimatisation period to the laboratory environment, the stress tests were conducted using 10 mean‐size adult individuals per replicate; except in two experiments when eight and nine individuals were used due to a lack of individuals (i.e. in a temperature—pCO_2_ experiment with *Mytilus* sp. from Schleimünde and a temperature—salinity experiment with *G. locusta* from Schleimünde, respectively). The experimental individuals were transferred from the acclimation conditions to the experimental conditions without gradual adjustments (see Table 1 and Table S1). Three replicates were conducted for each treatment. Mussel experiments were performed in 5 L aquaria, whereas those for gammarids were in 2 L aquaria containing artificial habitat structures to provide complexity of habitat. One aquarium represented one replicate per treatment. The experiments lasted for 30 days, with mortality checked daily. At that time, dead individuals, moulted exoskeletons (in the case of gammarids), and newly emerged juveniles (in the case of gammarids) were removed. Light and feeding regimes were kept the same as during the acclimation period. The experimental conditions and treatments differed among species/experiments, but analyses were performed within species (i.e. comparisons were made among intraspecific populations per experiment), and therefore our approach was not confounded by these disparities.

In the first type of experiment, that is, temperature—pCO_2_ experiments, the aquaria were placed in water baths at the desired temperature, with pCO_2_ levels being randomly assigned within each water bath, and water continuously infused with its determined pCO_2_ level using an air stone. The water used to fill the tanks was filtered from the surrounding area of the institute (through a 5 μm filter). The aquaria were sealed using a plastic cover to stabilise the pCO_2_ levels. Throughout the experiments, the salinity was maintained at the ambient condition for each species/population to reduce any additional stress. Temperature, pH (WTW pH 3110 with a SenTix 81 pH electrode), and salinity (WTW Cond 3110 with a Tetracon 325 probe) were recorded daily. Every 10 days, 50% of the water was exchanged with previously prepared water to ensure the desired temperature and pCO_2_ conditions were maintained. In the second type of experiment, that is temperature—salinity, the aquaria were placed in water baths at the desired temperature, with salinity levels being randomly assigned within each water bath. The aquaria were constantly aerated. Salinity and temperature were checked daily, with a 50% water change done every 10 days using previously prepared water to ensure the desired salinity and temperature conditions.

### 2.3 Statistical Analyses and Visualisation

Mortality curves were constructed for each population for each treatment, described by the equation (Briski et al. 2008, 2011):

(1) $$y=100/\left[1+e^{-Z\left(t-Q\right)}\right]$$

where *Z* is the slope of the mortality rate, *t* is time and *Q* is the onset of mortality. All curves were constructed using S‐Plus 6.1 (S‐Plus 6.1, 2002; Insightful Corp., Seattle, WA, USA). Raw data supporting the findings of this study is available at Dryad under doi: https://doi.org/10.5061/dryad.3tx95x6qx.

For each species and experiment separately, generalised linear models with binomial error distributions and log links were used to examine survival rates among populations, temperatures, and either salinities or pCO_2_ levels at day‐15 (mid‐point) and day‐30 (end‐point). Accordingly, 12 models were built in total (3 species × 2 experimental contexts [temperature + pCO_2_/salinity] × 2 time points). Non‐significant terms were removed backward and stepwise to obtain the most parsimonious models. In cases of complete separation (i.e. total survival or mortality in an experimental group), bias reductions were employed to fit the model to the data (Kosmidis 2020). Residuals were checked for overdispersion via comparisons of simulated and observed residual distributions (Hartig 2020). Analysis of deviance with type‐3 sums of squares was used to compute coefficients where there were more than two populations in a given model (i.e. models considering *G. salinus* ). Tukey comparisons were used to examine significant effects and interactions pairwise post hoc (Lenth 2020). Significance was inferred at *p* < 0.05, and generalised linear models were fit in R (R Core Development Team 2022).

## 3 Results

### 3.1 *Mytilus* Sp.

**FIGURE 2:**
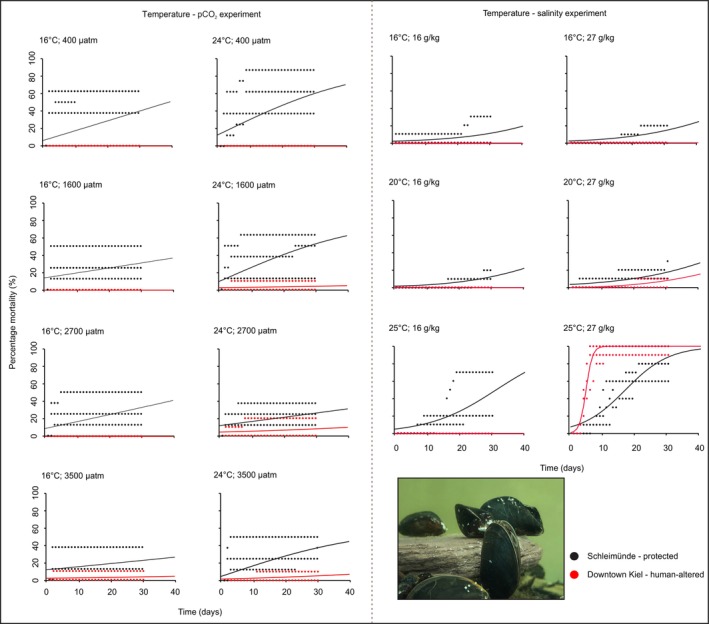
Percentage mortality for *Mytilus* sp. from protected and human‐altered habitats for the temperature‐pCO_2_ experiment (left) and the temperature‐salinity experiment (right).

In response to temperature and pCO_2_ gradients, significant differences for *Mytilus* sp. were observed between populations from human‐altered and protected habitats (Table S2), across both time points (Figure 2). Mortality in the population from the human‐altered habitat tended to peak at the highest pCO_2_ level, while mortality in the population from protected habitat peaked at the lowest pCO_2_ levels, driving a significant two‐way interaction between population and pCO_2_ (Table S2). Significant population‐level differences were also observed between groups from human‐altered and protected habitats in response to the experimental temperature and salinity gradients (Table S2), with the protected site population exhibiting greater mortality in all groups at both time points—except at the highest temperature and salinity (Figure 2). In that treatment, there was a much more rapid increase in mortality in the population from the human‐altered habitat, where it overtook the population from the protected habitat, as evidenced by a significant three‐way interaction term (Table S2).

#### 3.1.1 *Gammarus locusta*

**FIGURE 3:**
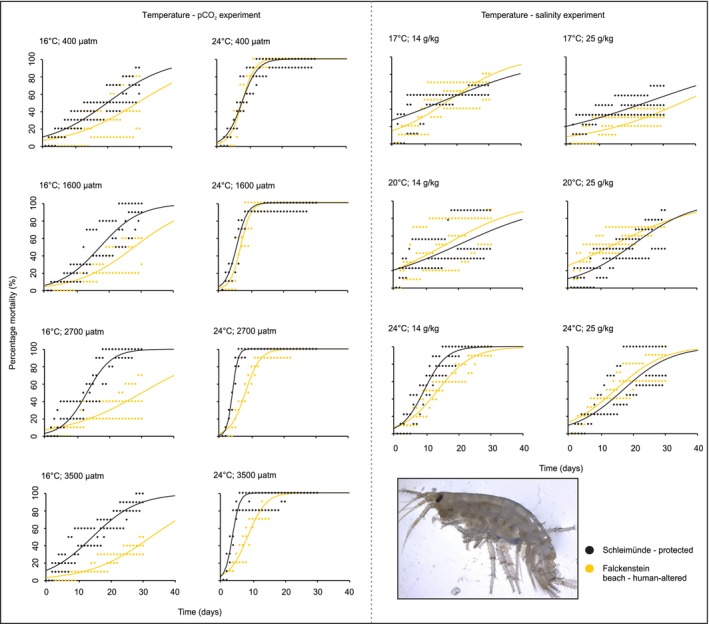
Percentage mortality for *Gammarus locusta* from protected and human‐altered habitats for the temperature‐pCO_2_ experiment (left) and the temperature‐salinity experiment (right).

In response to temperature and pCO_2_ gradients, population‐level responses by *G. locusta* were significantly influenced by pCO_2_ at both time points (Table S3), with the population from the protected habitat tending to exhibit higher mortality (Figure 3). Mortality rates of the population from human‐altered habitat tended to decrease with increasing pCO_2_, whereas the opposite was observed for the population from protected habitat (Figure 3). Therefore, population‐level mortalities were most divergent at the highest pCO_2_ levels. In contrast, no significant inter‐population differences were observed between those from protected and human‐altered habitats across temperature and salinity groups at either time point (Table S3). For both populations, mortality significantly increased with temperature and with falling salinity (Figure 3).

#### 3.1.2 *Gammarus salinus*

**FIGURE 4:**
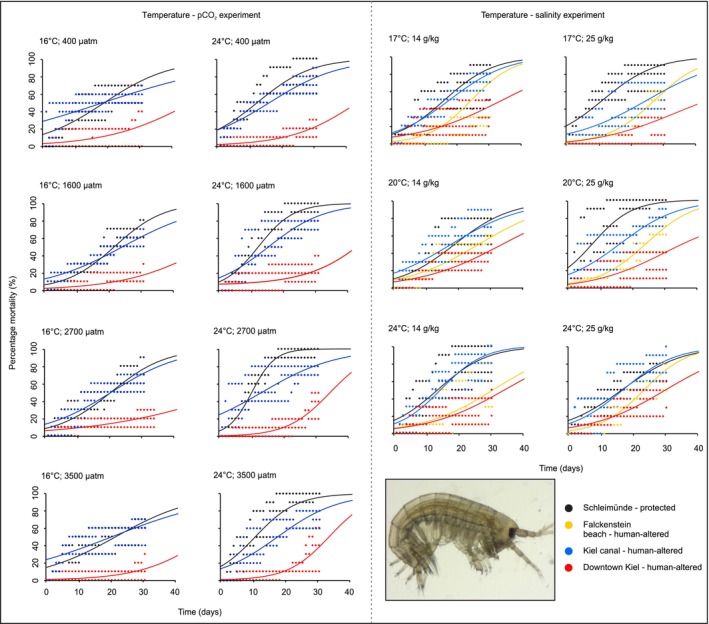
Percentage mortality for *Gammarus salinus* from protected and human‐altered habitats for the temperature‐pCO_2_ experiment (left) and the temperature‐salinity experiment (right).

In the temperature and pCO_2_ experiments, *G. salinus* exhibited significant differences according to temperature and population in interaction at day‐15 independently of pCO_2_ (Table S4). Mortality rates at day‐15 in the population from the most impacted human‐altered habitat (i.e. downtown Kiel) decreased with warming, while the other two populations (i.e. Kiel canal and Falckenstein beach) exhibited higher mortality with greater temperature (Figure 4). At day‐30, a significant three‐way interaction emerged, whereby differences between populations from the most impacted human‐altered habitat (i.e. downtown Kiel) and less impacted (i.e. Kiel canal) and protected habitats (i.e. Schleimünde) were greatest at the lower temperature and higher pCO_2_ levels (Table S4 and Figure 4). In the salinity and temperature experiments, inter‐population responses were influenced by temperature and salinity in interaction at day‐15, but not at day‐30 (Table S4). Mortality rates of all populations except the one from the protected habitat (i.e. Schleimünde) increased with greater temperature and at the lower salinity at day‐15. These differences were less obvious by day‐30, with mortality rates for human‐altered and protected habitat population differences more consistent (Figure 4). The population from the most impacted human‐altered habitat (i.e. downtown Kiel) and that from one of the less impacted (i.e. Falckenstein beach) had significantly lower mortality, with differences most pronounced between populations from those sites and populations from the other less impacted (i.e. Kiel canal) and protected habitats (i.e. Schleimünde) at the highest temperature and lower salinity. The population from the most impacted human‐altered habitat had significantly lower mortality than all other populations overall.

## 4 Discussion

Stresses associated with urban areas are pervasive, however, empirical testing of hypotheses linked to adaptation to anthropogenically modified habitats has remained scarce. Our study tested the performance of different populations of the same species of mussels and gammarids inhabiting impacted and protected habitats, and demonstrated that populations from impacted habitats typically performed better under a broad range of environmental conditions than those from the protected habitat. The populations from protected habitat were consistently less robust, or plastic, to multiple anthropogenic stressors. Based on stress performance and phenotypic plasticity, our results indicate that populations in urban, impacted habitats are adapting to life in anthropogenic environments.

Our study determined distinct differences between populations from impacted and protected habitats, with impacted environments producing populations more tolerant to multiple abiotic stressors. However, there was also an apparent stress response gradient within populations from urban areas of differing degrees of habitat alteration. This suggests that the development of resistance along anthropogenic gradients is continuous and not binary. Indeed, in the case of both gammarid species, the outer locations in the Kiel fjord at both the Falckenstein beach and Kiel canal sites produced populations less robust to stressed environments, with *G. salinus* exhibiting the highest tolerance at the most impacted and innermost downtown Kiel site. Falckenstein beach is sandy, lacking concrete and areas of altered habitat, however, the location is still exposed to frequent shipping transits throughout the year and the tourist industry in summer months. Kiel canal is located deeper in the Kiel fjord and contains a large amount of concrete as well as ship traffic. The most impacted site, downtown Kiel, is a primarily concrete artificial habitat, exposed to diverse anthropogenic impacts from the shipping industry, high heavy metal concentrations, as well as light and noise pollution and tourism (LLUR 2001; Nikulina, Polovodova, and Schönfeld 2008). Consequently, while specific environmental conditions driving adaptation require elucidation at these sites, it seems that the protected nature of a habitat does not play the main role in shaping the robustness of taxa. Rather, resistance is determined by the intensity of stressors present in a habitat, likely linked to the distance of the habitat from our cities and industries, and possibly the potential for taxa to move to surrounding refugia. While we made these inferences based on co‐occuring temperature, pCO_2_, and salinity stressors, which are pervasive in the Baltic Sea context, we acknowledge that other stressors linked to different forms of pollution (e.g. noise, light, heavy metals) require examination. Nevertheless, cross‐tolerance among heterologous stressors is possible (Todgham, Schulte, and Iwama 2005), with the ability of one stressor to increase tolerance to a second heterologous stressor, and therefore our stressor selection could approximate adaptive responses to other stressors.

As we did not test the mussel population from Falckenstein beach or the Kiel canal, but only from downtown Kiel, our study cannot show if mussels from these ‘intermediate’ impacted sites demonstrate the same pattern as gammarids. Nevertheless, mussels are sessile filter feeders and once their larvae are settled on a substrate, it is almost impossible for individuals to move further to more hospitable areas and seek refugia (Ruppert, Fox, and Barnes 2003). In contrast, gammarids are mobile detrivores that can actively retreat to less hostile environments when needed (Ruppert, Fox, and Barnes 2004; Gerhardt, Bloor, and Mills 2011). Therefore, we speculate that the mussel population from Falckenstein beach might have been more robust to stressors than the population from the protected habitat. We would speculate the same for other sessile organisms, such as macroalgae and seagrass. We encourage future studies to test our assumptions and assess taxonomic differences in stress resistance and adaptation among taxonomic groups across varyingly impacted habitats.

Occurrences of anthropogenically impacted populations underperforming individuals from the protected site were rare. However, in the highest temperature—salinity treatment, the population of *Mytilus* sp. from the impacted habitat exhibited a significantly faster mortality rate than the population from the protected habitat. As all replicates demonstrated similarly high mortality rates, we believe that there was no laboratory effect causing this rapid mortality of the population, but it was probably due to the combination of stressors being too impactful and exceeding the threshold of the population's stress tolerance. Similarly, in the case of *G. locusta* in the combination of the higher temperature (i.e. 24°C) with all pCO_2_ levels, the mortality of both populations was remarkably fast, with no survival displayed after 2 weeks. These examples further support population‐level nuances while also highlighting that even populations from anthropogenically‐stressed habitats are susceptible to abiotic limits. Hostile environments place extra pressure on organisms, as modifications from optimal environments require additional energetic costs because of, for example a higher rate of osmoregulation in suboptimal salinity regimes and an increase in metabolic rate with elevated temperatures, leading to a decrease in organisms' fitness (Gillooly et al. 2001; Evens 2008; Pörtner 2010; Bruno, Carr, and O'Connor 2015; Rivera‐Ingraham and Lignot 2017). In the case of highly hostile environments where the effects of stressors go beyond the threshold of the population's stress tolerance, those additional energetic costs are disproportionately high, and eventually lead to mortality (Gillooly et al. 2001; Evens 2008; Pörtner 2010; Bruno, Carr, and O'Connor 2015; Rivera‐Ingraham and Lignot 2017). Future research should elucidate whether gradual acclimatisation to these stressors can mediate levels of mortality within the laboratory through comparison with acute stress exposures. The resilience to the degree of variability in environmental stressors over time should also be examined among populations (Morón Lugo et al. 2020).

Furthermore, the ‘anthropogenically induced adaptation to invade’ (AIAI) hypothesis and urban evolution concept propose that contemporary adaptation of populations to human‐altered habitats (e.g. cities, shipping ports, fragmented urban habitats) within the native range can promote establishment success and increase levels of impact in non‐native regions (Hufbauer et al. 2012; Borden and Flory 2021; Perry and Gottert 2024). Indeed, human‐altered habitats have relatively similar environmental conditions globally and are increasingly prevalent with human population growth, especially in cities (Grimm et al. 2008). Moreover, most long‐distance transport happens between two urban habitats (e.g. between two shipping ports; Hulme 2009; Kaluza et al. 2010; Sardain, Sardain, and Leung 2019), and thus, the presence of adapted populations in the human‐altered habitats can experience increased transport probability, higher chances of surviving the harsh transport conditions, and a reduced need for further adaptation in the non‐native regions following introduction (Hufbauer et al. 2012; Lockwood, Hoopes, and Marchetti 2013; Briski et al. 2013, 2014, 2018). Finally, due to broader phenotypic plasticity of those populations, they would also have better chances for further adaptation and evolution in new habitats, particularly if new habitats are more variable and different from their native ones (Buczkowski 2010; Dlugosch et al. 2015; Cadotte et al. 2017; van Kleunen et al. 2018; Westneat et al. 2019; Haubrock et al. 2021). Therefore, adaptations to anthropogenic and urban stressors could contribute substantially to invasion success of non‐native species across all stages of the invasion process—uptake in a native region, transport, introduction, establishment and spread/impact.

These altered ecosystems can also act as experimental ‘time machines’ for studying adaptation potential to predicted future global changes, as these habitats, for example, are often warmer and have higher levels of CO_2_ than surrounding natural areas (Grimm et al. 2008; Lahr, Dunn, and Frank 2018). In our study area, Kiel fjord, for example, is characterised by strong seasonal pCO_2_, variability (Thomsen et al. 2012). Similarly, large estuaries, which are heavily inhabited by humans and contain numerous shipping ports, such as the Saint Lawrence estuary in Canada, or the Chesapeake Bay and Mississippi and Atchafalaya River estuaries in the US, reveal similar patterns (Dinauer and Mucci 2017; Lohrenz et al. 2018; Chen et al. 2020). Moreover, those locations are also characterised by huge salinity and temperature variations associated with anthropogenic environmental change (Casties, Seebens, and Briski 2016; Pansch et al. 2018; Hinson et al. 2022; Kazanavičiūtė et al. 2024). On land, a similar pattern could be observed in impacted and protected sites between stressors such as temperature (i.e. urban ‘heat islands’) and pollutants (Borden and Flory 2021).

Following a multiple‐stressor framework with factorial stressor arrangements, our experiments demonstrated greater resistance to these stressors and higher phenotypic plasticity by the populations from the impacted habitats, particularly those more closely located to the city and experiencing the greatest disturbance connected to, for instance, the shipping industry. We demonstrate that populations inhabiting urban environments not only endure human‐altered habitat conditions, but they are also more resistant to diverse stressors associated with global environmental change. Therefore, we speculate that those populations would be suitable for genetic rescue to increase population fitness through the introduction of new genetic material to endangered populations/species (Tallmon, Luikart, and Waples 2004; Whiteley et al. 2015; Fitzpatrick et al. 2023). However, caution is needed in translocation of these populations, as they have a higher probability of establishing in non‐native areas, especially other urban environments (Hufbauer et al. 2012; Borden and Flory 2021; Perry and Gottert 2024). Finally, additional studies testing resistance to various stressors of diverse taxa from multiple geographic locations and environments are needed to confirm the generality of our findings.

## Author Contributions

**Elizabeta Briski:** conceptualization. **Elizabeta Briski, Louisa Langrehr, Syrmalenia G. Kotronaki, Alena Sidow, Cindy Giselle Martinez Reyes, Antonios Geropoulos, Gregor Steffen, Nora Theurich, James W.E. Dickey, Jasmin C. Hütt and Ross N. Cuthbert:** investigation. **Elizabeta Briski, Phillip J. Haubrock, Ismael Soto, Antonín Kouba and Ross N. Cuthbert:** formal analysis and visualisation. **Elizabeta Briski and Ross N. Cuthbert:** writing – original draft. All authors: writing – review and editing. **Ross N. Cuthbert, James W.E. Dickey and Elizabeta Briski:** funding acquisition.

## Ethics Statement

Ethical approval was not required for the nature of this work.

## Conflicts of Interest

The authors declare no conflicts of interest.

### Peer Review

The peer review history for this article is available at https://www.webofscience.com/api/gateway/wos/peer‐review/10.1111/ele.70074.

## Supporting information


Table S1.

## Data Availability

Raw data supporting the findings of this study is available at Drayad: https://doi.org/10.5061/dryad.3tx95x6qx and R code used for all of the GLMs at Zenedo: https://doi.org/10.5281/zenodo.14616924.
