# Supplementary material for: Urban Environments Promote Adaptation to Multiple Stressors
Source: Ecol Lett. 2025 Feb 19;28(2):e70074. doi: 10.1111/ele.70074 (PMC11836597; doi:10.1111/ele.70074)
Supplement: Supplementary file 1 — Table S1. [file ELE-28-0-s001.docx]

**Urban environments promote adaptation to multiple stressors**

**SUPPORTING INFORMATION**

Elizabeta Briski^1^ (ebriski@geomar.de), Louisa Langrehr^1,2^(Louisa.Langrehr@gmx.net), Syrmalenia G. Kotronaki^1,3^(sirmalenia.k@gmail.com), Alena Sidow^1^(alena.sidow@web.de), Cindy Giselle Martinez Reyes^1^(cgmartinez96@gmail.com), Antonios Geropoulos^4^(bio2822@hotmail.com), Gregor Steffen^1^(phat@gmx.com), Nora Theurich^1,5^(nora.theurich@gmail.com), James W.E. Dickey^1,6,7,8^(jamesdickey03@gmail.com), Jasmin C. Hütt^1^(jashuett@gmail.com), Phillip J. Haubrock^,9,10,11^(phillip.haubrock@senckenberg.de), Ismael Soto^9^(isma-sa@hotmail.com), Antonín Kouba^9^(akouba@frov.jcu.cz), and Ross N. Cuthbert^1,12^(r.cuthbert@qub.ac.uk)

# ^1^GEOMAR Helmholtz-Zentrum für Ozeanforschung Kiel, Wischhofstraße 1-3, 24148 Kiel, Germany

^2^Carl von Ossietzky Universität Oldenburg, Ammerländer Heerstraße 114-118, 26129 Oldenburg, Germany

^3^ Department of Biological Sciences, Lehigh University, Bethlehem PA 18015, USA

^4^ Faculty of Science and Technology, Biology Department University of Crete, Marine Ecology Lab, Vasilika Vouton, 70013 Heraklio, Crete, Greece, P.O.Box 2208

^5^Christian-Albrechts-Universität zu Kiel Christian-Albrechts-Platz 4, 24118 Kiel, Germany

^6^Berlin-Brandenburg Institute of Advanced Biodiversity Research, 14195 Berlin, Germany

^7^Freie Universität Berlin, Institute of Biology, 14195 Berlin, Germany

^8^Leibniz Institute of Freshwater Ecology and Inland Fisheries (IGB), 12587 Berlin, Germany

^9^University of South Bohemia in České Budějovice, Faculty of Fisheries and Protection of Waters, South Bohemian Research Center of Aquaculture and Biodiversity of Hydrocenoses, Zátiší 728/II, 389 25 Vodňany, Czech Republic

^10^Senckenberg Research Institute and Natural History Museum Frankfurt, Department of River Ecology and Conservation, Gelnhausen, Germany

^11^Center for Applied Mathematics and Bioinformatics, Department of Mathematics and Natural Sciences, Gulf University for Science and Technology, Hawally, Kuwait

^12^Institute for Global Food Security, School of Biological Sciences, Queen’s University Belfast, 19 Chlorine Gardens, Belfast BT9 5DL, Northern Ireland, UK

Table S1 *Mytilus* sp., *Gammarus locusta* and *G. salinus* population source locations, sampling dates, and abiotic conditions during sampling from protected and human-altered habitats.

| Temperature – pCO_2_ experiments | | | | | | | | | | |
| --- | --- | --- | --- | --- | --- | --- | --- | --- | --- | --- |
|  | Protected habitat | | | | | Human altered habitats | | | | |
| Species | Location | Coordinates | Sampling date | Temperature (°C) | Salinity (g/kg) | Location | Coordinates | Sampling date | Temperature (°C) | Salinity (g/kg) |
| *Mytilus* sp. | Schleimünde | 54°40'33.0"N 10°01'48.0"E | December 2020 | 12 | 17 | Downtown Kiel | 54°19'45.7"N 10°08'55.7"E | November 2020 | 12 | 17 |
| *Gammarus locusta* | Schleimünde | 54°40'33.0"N 10°01'48.0"E | May 2019 | 16 | 16 | Falckenstein beach | 54°23'36."N 10°11'21.4"E | April 2019 | 15 | 16 |
| *Gammarus salinus* | Schleimünde | 54°40'33.0"N 10°01'48.0"E | June 2019 | 16 | 17 | Downtown Kiel | 54°19'45.7"N 10°08'55.7"E | July 2019 | 16 | 16 |
|  |  |  |  |  |  | Kiel canal | 54°22'55.3"N 10°09'43.6"E | August 2020 | 15 | 16 |
| Salinity – temperature experiments | | | | | | | | | | |
|  | Protected habitat | | | | | Human altered habitats | | | | |
| Species | Location | Coordinates | Sampling date | Temperature (°C) | Salinity (g/kg) | Location | Coordinates | Sampling date | Temperature (°C) | Salinity (g/kg) |
| *Mytilus* sp. | Schleimünde | 54°40'33.0"N 10°01'48.0"E | September 2016 | 18 | 16 | Downtown Kiel | 54°19'45.7"N 10°08'55.7"E | October 2016 | 16 | 16 |
| *Gammarus locusta* | Schleimünde | 54°40'33.0"N 10°01'48.0"E | July 2020 | 16 | 14 | Falckenstein beach | 54°23'36."N 10°11'21.4"E | July 2020 | 18 | 14 |
| *Gammarus salinus* | Schleimünde | 54°40'33.0"N 10°01'48.0"E | May 2016 | 18 | 14 | Downtown Kiel | 54°19'45.7"N 10°08'55.7"E | June 2021 | 15 | 13 |
|  |  |  |  |  |  | Kiel canal | 54°22'55.3"N 10°09'43.6"E | September 2020 | 17 | 14 |
|  |  |  |  |  |  | Falckenstein beach | 54°23'36."N 10°11'21.4"E | May 2016 | 15 | 16 |

**Table S2.** Generalized linear models (binomial) considering *Mytilus* sp. mortality as a function of population (Pop), temperature (Tem), and salinity (Sal) or pCO_2_ (Pco) at day-15 and day-30. Coefficients were obtained via backward, stepwise deletion. Significant terms are shown in bold (*p* < 0.05).

| Treatment | Day-15 | | | Day-30 | | |
| --- | --- | --- | --- | --- | --- | --- |
|  | Term | *z*-value | *p*-value | Term | *z*-value | *p*-value |
| (a) Temperature – pCO_2_ experiments | **Pop** | **4.196** | **< 0.001** | **Pop** | **4.151** | **< 0.001** |
|  | Tem | 0.865 | 0.387 | Tem | 1.141 | 0.254 |
|  | Pco | 0.334 | 0.738 | Pco | 0.454 | 0.650 |
|  | Pop:Tem | 1.018 | 0.309 | Pop:Tem | 0.929 | 0.353 |
|  | **Pop:Pco** | **2.146** | **0.032** | **Pop:Pco** | **2.029** | **0.042** |
|  | Tem:Pco | 0.730 | 0.465 | Tem:Pco | 0.338 | 0.735 |
|  | Pop:Tem:Pco | 0.245 | 0.806 | Pop:Tem:Pco | 0.352 | 0.725 |
| (b) Temperature – salinity experiments | Pop | 1.842 | 0.065 | Pop | 1.488 | 0.137 |
|  | **Tem** | **2.288** | **0.022** | **Tem** | **2.027** | **0.042** |
|  | **Sal** | **2.943** | **0.003** | **Sal** | **2.786** | **0.005** |
|  | **Pop:Tem** | **2.111** | **0.035** | Pop:Tem | 1.911 | 0.056 |
|  | **Pop:Sal** | **2.204** | **0.028** | **Pop:Sal** | **2.147** | **0.032** |
|  | **Tem:Sal** | **3.471** | **< 0.001** | **Tem:Sal** | **3.354** | **< 0.001** |
|  | **Pop:Tem:Sal** | **2.485** | **0.013** | **Pop:Tem:Sal** | **2.496** | **0.013** |

**Table S3.** Generalized linear models (binomial) considering *Gammarus locusta* mortality as a function of population (Pop), temperature (Tem), and salinity (Sal) or pCO_2_ (Pco) at day-5 and day-30. Coefficients were obtained via backward, stepwise deletion. Significant terms are shown in bold (*p* < 0.05).

| Treatment | Day-15 | | | Day-30 | | |
| --- | --- | --- | --- | --- | --- | --- |
|  | Term | *z*-value | *p*-value | Term | *z*-value | *p*-value |
| (a) Temperature – pCO_2_ experiments | Pop | 0.704 | 0.482 | Pop | 1.556 | 0.120 |
|  | **Tem** | **10.114** | **< 0.001** | **Tem** | **4.450** | **< 0.001** |
|  | Pco | 1.864 | 0.062 | **Pco** | **2.423** | **0.015** |
|  | Pop:Tem | 1.201 | 0.230 | Pop:Tem | 0.670 | 0.503 |
|  | **Pop:Pco** | **3.050** | **0.002** | **Pop:Pco** | **2.857** | **0.004** |
|  | Tem:Pco | 1.887 | 0.059 | **Tem:Pco** | **2.075** | **0.038** |
|  | Pop:Tem:Pco | 1.522 | 0.128 | Pop:Tem:Pco | 0.159 | 0.874 |
| (b) Temperature – salinity experiments | Pop | 0.417 | 0.676 | Pop | 0.080 | 0.936 |
|  | **Tem** | **2.652** | **0.008** | **Tem** | **5.737** | **< 0.001** |
|  | **Sal** | **2.072** | **0.038** | **Sal** | **2.482** | **0.013** |
|  | Pop:Tem | 0.117 | 0.907 | Pop:Tem | 0.522 | 0.601 |
|  | Pop:Sal | 0.439 | 0.661 | Pop:Sal | 0.873 | 0.382 |
|  | Tem:Sal | 0.012 | 0.990 | Tem:Sal | 0.014 | 0.989 |
|  | Pop:Tem:Sal | 1.906 | 0.057 | Pop:Tem:Sal | 1.110 | 0.267 |

**Table S4.** Generalized linear models (binomial) considering *Gammarus salinus* mortality as a function of population (Pop), temperature (Tem), and salinity (Sal) or pCO_2_ (Pco) day-15 and day-30. Coefficients were obtained via backward, stepwise deletion and analysis of deviance (LRT = likelihood ratio test chi-square statistic; df = degrees of freedom). Significant terms are shown in bold (*p* < 0.05).

| Treatment | Day-15 | | | Day-30 | | |  |
| --- | --- | --- | --- | --- | --- | --- | --- |
|  | Term | LRT (df) | *p*-value | Term | LRT (df) | *p*-value | |
| (a) Temperature – pCO_2_ experiments | Pop | 3.646 (2) | 0.161 | Pop | 1.863 (2) | 0.394 | |
|  | **Tem** | **4.754 (1)** | **0.029** | Tem | 0.082 (2) | 0.775 | |
|  | Pco | 1.513 (1) | 0.219 | **Pco** | **7.832 (1)** | **0.005** | |
|  | **Pop:Tem** | **6.757 (2)** | **0.034** | Pop:Tem | 3.510 (2) | 0.173 | |
|  | Pop:Pco | 4.014 (2) | 0.134 | Pop:Pco | 4.430 (2) | 0.109 | |
|  | Tem:Pco | 1.278 (1) | 0.258 | **Tem:Pco** | **10.035 (1)** | **0.002** | |
|  | Pop:Tem:Pco | 1.436 (2) | 0.488 | **Pop:Tem:Pco** | **6.324 (2)** | **0.042** | |
| (b) Temperature – salinity experiments | **Pop** | **8.717 (3)** | **0.033** | **Pop** | **96.506 (3)** | **< 0.001** | |
|  | Tem | 0.836 (1) | 0.361 | **Tem** | **9.104 (1)** | **0.003** | |
|  | Sal | 2.911 (1) | 0.088 | Sal | 0.333 (1) | 0.564 | |
|  | **Pop:Tem** | **8.901 (3)** | **0.031** | Pop:Tem | 4.366 (3) | 0.225 | |
|  | **Pop:Sal** | **14.366 (3)** | **0.002** | Pop:Sal | 5.671 (3) | 0.129 | |
|  | Tem:Sal | 2.134 (1) | 0.144 | Tem:Sal | 2.718 (1) | 0.099 | |
|  | **Pop:Tem:Sal** | **12.254 (3)** | **0.007** | Pop:Tem:Sal | 3.498 (3) | 0.321 | |
